# Scoring system predictive of survival for patients undergoing stereotactic body radiation therapy for liver tumors

**DOI:** 10.1186/1748-717X-7-148

**Published:** 2012-09-05

**Authors:** Marie-Adele S Kress, Brian T Collins, Sean P Collins, Anatoly Dritschilo, Gregory Gagnon, Keith Unger

**Affiliations:** 1 Department of Radiation Oncolog, Georgetown University Hospital, Lower Level Bles, 3800 Reservoir Road, Georgetown, N.W. Washington, D.C., 20007, USA

**Keywords:** Liver, Liver metastases, Hepatic metastases, Liver tumors, Stereotactic body radiation therapy, CyberKnife

## Abstract

**Background:**

Stereotactic body radiation therapy (SBRT) is an emerging treatment option for liver tumors. This study evaluated outcomes after SBRT to identify prognostic variables and to develop a novel scoring system predictive of survival.

**Methods:**

The medical records of 52 patients with a total of 85 liver lesions treated with SBRT from 2003 to 2010 were retrospectively reviewed. Twenty-four patients had 1 lesion; 27 had 2 or more. Thirteen lesions were primary tumors; 72 were metastases. Fiducials were placed in all patients prior to SBRT. The median prescribed dose was 30 Gy (range, 16 – 50 Gy) in a median of 3 fractions (range, 1–5).

**Results:**

With median follow-up of 11.3 months, median overall survival (OS) was 12.5 months, and 1 year OS was 50.8%. In 42 patients with radiographic follow up, 1 year local control was 74.8%. On univariate analysis, number of lesions (p = 0.0243) and active extralesional disease (p < 0.0001) were predictive of OS; Karnofsky Performance Status (KPS) approached statistical significance (p = 0.0606). A scoring system for predicting survival was developed by allocating 1 point for each of the three following factors: active extralesional disease, 2 or more lesions, and KPS ≤ 80%. Score was associated with OS (p < 0.0001). For scores of 0, 1, 2 and 3, median survival intervals were 34, 12.5, 7.6, and 2.8 months, respectively.

**Conclusions:**

SBRT offers a safe and feasible treatment option for liver tumors. A prognostic scoring system based on the number of liver lesions, activity of extralesional disease, and KPS predicts survival following SBRT and can be used as a guide for prospective validation and ultimately for treatment decision-making.

## Background

The liver is a common site for metastatic disease from multiple primary tumors, including colorectal, breast, and lung cancer. Historically, limited metastatic disease was managed with surgical resection, with 5-year survival up to 67% [1,2]. However, surgery is an option only for patients with limited disease and adequate performance status, which may be as few as 10-20% of patients with hepatic metastases [1,3-5]. Primary liver tumors are managed similarly, with resection providing the only potentially curative option. However, patients with metastatic, primary, or recurrent liver tumors not amenable to resection are candidates for local treatments including radiofrequency ablation (RFA), trans-arterial chemo-embolization (TACE), or stereotactic body radiation therapy (SBRT), which are increasingly used with the goal of achieving local control.

Modern, conventional radiation therapy (RT) has improved upon historical approaches by improving conformality and minimizing dose to normal liver [6]. However, due to respiratory motion and set-up uncertainty, even these modern techniques are limited in terms of their potential for dose-escalation and effective tumor control. SBRT has been employed for the treatment of inoperable, limited tumors in the lungs, brain, and other sites of oligometastatic or limited primary disease [7-10]. Recently, SBRT has begun to be used for the treatment of limited liver metastases [11-14].

Despite growing evidence supporting SBRT as safe and effective for local control of liver lesions, little is known regarding optimal patient selection for this treatment modality. In other sites of metastatic disease, algorithms or scoring systems have been developed to identify candidates for radiation therapy and to establish patients’ prognoses, such as the Recursive Partitioning Analysis or Diagnosis-Specific Graded Prognostic Assessment classes used in the treatment of patients with brain metastases [15,16].

In this study, we aimed to review clinical outcomes of patients treated with SBRT for liver lesions and to develop a novel scoring system to predict overall survival (OS) and to guide treatment decision-making.

## Methods

### Patient selection

This retrospective review was approved by the Institutional Review Board (IRB) of Georgetown University. Eight-five lesions in 52 consecutive patients treated with SBRT for liver metastases between November, 2004 and June, 2009 were identified from treatment records at Georgetown University Hospital’s Department of Radiation Oncology. No patients were considered candidates for surgical resection. Lesions were considered for treatment in any location within the liver, including right, left, and caudate lobes, as well as proximal to the porta hepatis. All histologies were included, including metastatic and primary liver tumors. Patients were included irrespective of prior treatment. Patients generally were excluded for inadequate hepatic function. Patients were generally seen in regular follow-up for clinical and radiologic assessments at the discretion of the treating radiation and medical oncologists every 3 to 6 months. Initial radiologic follow-up included contrast-enhanced CT scan, MRI, and/or PET/CT scan and was typically 2–4 months after completion of CyberKnife SBRT.

### SBRT planning and treatment

SBRT planning and treatment techniques have been detailed previously [17]. Three to five gold fiducials were placed in or near liver tumors under CT guidance (Best Medical, Springfield, VA). A treatment planning CT scan with slice thickness of 1 – 3 mm was obtained at least 5 days after fiducial placement. Patients were simulated in the supine position. Gross tumor volume (GTV) was delineated on the CT scan. Typically, margins of at least 3–5 mm were added to the GTV to form the clinical target volume (CTV) [18]. No additional margin was added to form the planning target volume (PTV). Adjacent critical structures were delineated.

All treatments were performed using the CyberKnife system and were planned using Multiplan treatment planning software. Radiation plans and prescriptions were developed using inverse-planning. Treatments were delivered using 6MV photons and were prescribed to an isodose line that provided adequate PTV coverage (> 95%). Synchrony Respiratory Tracking System was used to continuously track fiducial position and adjust for respiratory motion. Patients were treated to a median dose of 30 Gy (range: 16 – 50 Gy), in a median of 3 fractions (range: 1–5). A biologic equivalent dose (BED) was calculated for each fractionation scheme by the formula: BED_10_ = (Prescription dose) * (1+ (Dose per fraction / α/β)); where α/β is assumed to be 10.

### Data analysis

Treatment response was evaluated by serial CT, PET, and/or MRI scans. Estimates of initial treatment response were determined using the Response Evaluation Criteria in Solid Tumors (RECIST) [19]. Local control was defined as no evidence of tumor growth of the treated lesion. Actuarial survival and local control rates were evaluated by the Kaplan-Meier method. Univariate analysis (UVA) was performed with the logrank test; multivariate analysis (MVA) was performed using Cox proportional-hazards regression. Based on the UVA analysis a prognostic scoring system for survival was devised whereby the presence of the following increased the score by one point each: presence of active systemic disease, 2 or more liver tumors, and KPS ≤ 80. Toxicities were evaluated according to the Common Terminology Criteria for Adverse Events (CTCAE), Version 4.0 [20]. Toxicities occurring less than or equal to 3 months following SBRT were considered acute, while toxicities occurring after 3 months were considered late.

## Results

### Patient and lesion characteristics

Patient characteristics are noted in Table 1. The median age at treatment was 56. Eighty-nine percent of lesions had not been treated in the past. The median Karnofsky Performance Status (KPS) at the time of treatment was 90 (range, 50–90). Thirty-six patients had good KPS (90-100%), while 14 patients had KPS of 80 or less, with only 2 patients demonstrating poor KPS (50-60%). Twenty-four patients only had one liver lesion at the time of SBRT, while 9 had two lesions, and 18 had three or more. Twenty-four patients (46%) had active systemic, extrahepatic disease at the time of SBRT, while the remainder did not.

**Table 1:** Patient characteristics

**Characteristic**	**n**
**Total patients (*n*)**	52
**Total lesions**	85
**Sex (*n)*(per patient)**	
M	29
F	23
**Age (y) (per patient)**	
Median	56
Range	37-91
**Number of liver lesions (per patient)**	
One	24
Two	9
Three or more	18
**Karnofsky Performance Status (KPS)**	
Median	90
Range	50-100
**Status of systemic disease**	
Active	24
Inactive	28

Lesion characteristics are noted in Table 2, and dosimetric parameters are presented in Table 3. Most lesions were treated using a fractionated approach, with a median of 3 fractions (range, 1–5). The BED_10_ varied widely, with a median effective dose of 60 Gy (range, 28 – 113 Gy), which is equivalent to a median dose of 50 Gy (range, 23 – 94 Gy) using standard (2 Gy) fractionation. Lesion size also varied greatly, with a median treatment volume of 32 cm^3^.

**Table 2:** Lesion characteristics

**Characteristic**	**n**
**Location of lesion (per lesion)**	
Right lobe	56
Left lobe	20
Porta hepatis	3
Caudate	4
**Prior therapy to treated lesion**	
Prior radiation	2
Chemo-embolization	3
Radiofrequency ablation	3
Surgery	1
**Histology**	
Colorectal	14
Pancreatic	12
Ovarian	12
Leiomysarcoma	11
Cholangiocarcinoma	11
Hepatocellular	5
Squamous cell carcinoma of the head and neck	4
Malignant meningioma	2
Adenocarcinoma of unknown primary	2
Transitional cell	2
Non-small-cell lung cancer	2
Fibrous histiocytoma	2
Breast	2
Prostate	2
Esophageal	1
Uterine	1

**Table 3:** Dosimetric parameters

**Characteristic**	**n**
**Tumor volume, cm**^**3**^	
Mean	83
Median	32
Range	0.37-643.5
**Prescribed dose, Gy**	
Mean	28
Median	30
Range	16-50
**Number of fractions**	
Median	3
Range	1-5
Median dose per fraction	10 Gy
**BED**_**10**_**, Gy**	
Mean	56
Median	60
Range	28-113
**Prescription isodose line, %**	
Mean	75
Median	75
Range	60-90
**Conformality index**	
Mean	1.70
Median	1.56
Range	1.15-3.12

### Clinical outcomes

**Figure 1:**
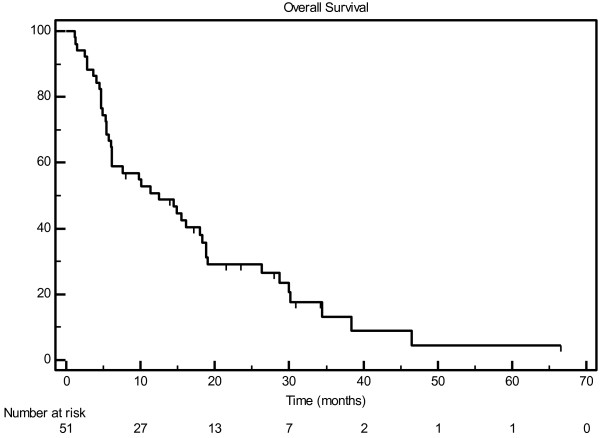
Overall survival by patient.

**Figure 2:**
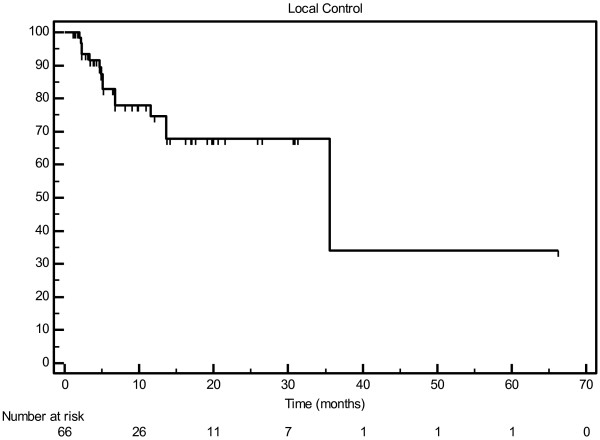
Local control by lesion.

With a median follow-up of 11.3 months (range, 1–67 months), the median actuarial overall survival was 12.5 months, with a one-year actuarial survival of 50.8% (Figure 1). Twelve patients with a total of 14 lesions had no radiographic follow-up. Among those with post-treatment imaging, local control was excellent, with median time to failure of 35.5 months and actuarial 1-year local control of 74.8% (Figure 2).

BED_10_ > 75 Gy, primary versus metastatic lesion, target volume (less than 32 vs. 32 or more), and histology (colorectal cancer vs. all others) were analyzed for correlation with local control. Of these variables, only small volume was associated with improved local control on univariate (p = 0.0449) and multivariate (p = 0.0295) analyses.

**Figure 3:**
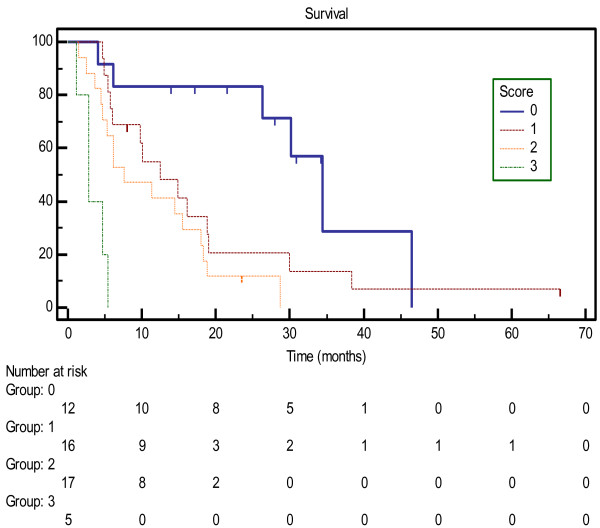
Survival by prognostic score.

On (UVA), reported in Table 4, the number of lesions (1 vs. 2 or more) (p = 0.0243) and active systemic disease (p < 0.0001) were predictive of survival. Status of tumor as primary vs. metastasis was also evaluated, but was not found to be associated with survival (Table 4). KPS (80 or less vs. 90–100) approached statistical significance (p = 0.0606). A scoring system was developed, whereby each of the following factors increased the patient’s score by one point: 2 or more lesions, active systemic disease, and KPS of 80 or less. As shown in Figure 3, this scoring system was predictive of OS. Median survival by score was 34.4 months, 12.5 months, 7.6 months, and 2.8 months for scores of 0, 1, 2, and 3, respectively. On MVA, patient score was the only factor predictive of survival (p < 0.0001).

**Table 4:** Univariate and multivariate analysis for overall survival

**Univariate analysis**	***p***
Number of lesions	
1 vs. 2 or more	0.0243
Active Systemic Disease	<0.0001
Karnofsky Performance Status (KPS)	
80 or less vs. 90-100	0.0606
Primary vs. metastasis	0.2755
Histology	0.6879
**Multivariate analysis**	***p***
Score	<0.0001
Primary vs. metastasis	0.6423
Histology	0.5644

### Toxicity

Overall, 11.5% of patients experienced grades 3/4 acute toxicities. Grade 4 elevation in bilirubin was experienced by one patient, followed by early death due to tumor progression. This patient also had grades 1, 2, and 3 elevations in alanine aminotransferase (ALT), alkaline phosphatase, and aspartate aminiotransferase (AST), respsectively. There was one grade 3 toxicity due to acalculous cholecystitis that required percutaneous drainage and treatment with antibiotics. There were 4 additional grade 3 elevations of bilirubin, and one of those patients also had a grade 3 elevation of alkaline phosphatase. Grade 1–2 toxicities were common and included right upper quadrant abdominal pain, anorexia, weight loss, liver enzyme elevations, fatigue, nausea, vomiting, and gastritis. No patients required treatment interruptions due to toxicity.

## Discussion

Stereotactic body radiation therapy (SBRT) has been demonstrated to be feasible and safe for treatment of liver tumors in multiple retrospective and a few prospective series [11-14,21,22]. A single-institution Phase I study established the safety of single fraction SBRT with no grade 3 or higher toxicities, 1-year local control of 77%, and median overall survival of 28.6 months [23]. A multi-institutional Phase I/II study demonstrated limited toxicity (2% grade 3 or higher) and excellent in-field local control at two years (92%), which improved with small lesion size (100% at two years), with a median survival of 20.5 years [24]. A larger Phase I-II study did not reach dose-limiting toxicity, with a total of 13% of patients with grades 3–4 toxicity, and no grade 5 toxicity; 1-year local control was 71%, with 17.6 months median overall survival [25].

Our study compares favorably with these prospective and retrospective studies. Although the lower median survival may relate to the significant number of patients with compromised performance status and active extrahepatic disease reflected in this series, our 1-year local control rate of 74.8% is comparable with published series. Retrospective reports have demonstrated 1 to 2-year local control ranging from 57-92%, with median survival ranging from 14.5-34 months [21,22,26-28]. However, this heterogeneous group of patients may be expected to have widely variable clinical outcomes.

Despite the growing interest in using SBRT to treat hepatic metastases, little is known about optimal patient selection or prognosis. In the setting of high long-term local control rates, it becomes of paramount importance to select patients who may optimally benefit from local control and omit those who would be unlikely to clinically benefit from treatment.

Clinical scoring systems have been used to prognosticate outcomes for patients with metastatic disease in other sites. One prominent example is the use of Recursive Partitioning Analysis (RPA) or Diagnosis-Specific Graded Prognostic Assessment (DS-GPA) for patients with brain metastases [15,16]. RPA classes were determined by Karnofsky Performance Status (KPS), age, and status of primary and extracranial metastases [15]. DS-GPA factors vary by primary diagnosis, but all scores incorporate KPS, while scores for non-small cell lung cancer (NSCLC), small cell lung cancer (SCLC), melanoma, and renal cell cancer incorporate the number of brain lesions, while NSCLC and SCLC also incorporate the status of extracranial metastases [16].

Surgical series have examined pre- and peri-operative prognostic factors in the setting of hepatic metastectomy. One study demonstrated that predictors of long-term outcome included extrahepatic disease status, number and size of hepatic tumors [29]. The authors of this study demonstrated that a preoperative scoring system combining carcinoembryonic antigen level, number and size of tumors, disease-free interval from primary to metastases and lymph node status of primary was predictive of survival [29]. A similar published study observed that 10 separate factors were associated with surgical outcome from hepatectomy. These factors included patient age, tumor histology, length of disease-free interval between treatment of primary tumor and metastases, presence of extrahepatic disease, response to pre-operative chemotherapy, time period of treatment, and surgical factors [30]. The authors also developed a risk model, based on these 10 characteristics, that was correlated with estimated 5-year survival [30]. Our study utilizes some of the same factors used in these scoring systems, such as performance status and extralesional disease status, but combines them in a simple, clinically accessible model. However, one limitation of our scoring system is that it has been developed using only a retrospective data set. As a result, this system’s utility in evaluating candidates for hepatic SBRT warrants validation with a prospective cohort and for individual disease subtypes.

Other local therapy options have been evaluated for use in the setting of limited hepatic metastases, one of the most common of which is radiofrequency ablation (RFA). As with SBRT, outcomes following RFA have been shown to be related to the number and size of hepatic tumors [31]. Previous studies have demonstrated that the extrahepatic disease status and primary tumor site correlated with survival following liver SBRT [24,32].

From these historical scoring systems and review of outcomes following local therapies, including SBRT, it is evident that there may be many significant factors that relate to survival in the setting of treating patients with limited metastatic disease. Performance status, status of extralesional disease, and number of lesions, the three factors utilized in our novel scoring system, are commonly used in the systems discussed above. The novel scoring system presented in this paper represents a potential paradigm-shift in patient selection for SBRT. However, this system is hypothesis-generating since it is based on retrospective data. This system needs to be validated with a prospective patient cohort and matched with information regarding patient-reported quality of life outcomes and step-wise approaches to additional local, regional, and systemic therapy.

## Conclusions

SBRT is a safe and feasible treatment option for liver tumors. A prognostic scoring system that includes the number of liver lesions, activity of extralesional disease, and KPS predicts overall survival following liver SBRT. This system will benefit from further refinement with a prospective cohort to add specificity regarding prognoses for individual pathologic subtypes and in the presence of combination therapy.

## Abbreviations

ALT: Alanine aminotransferase; AST: Aspartate aminotransferase; BED: Biologic equivalent dose; CTCAE: Common Terminology Criteria for Adverse Events; CTV: Clinical target volume; GTV: Gross tumor volume; IRB: Institutional Review Board; KPS: Karnofsky performance status; NSCLC: Non-small cell lung cancer; PTV: Planning target volume; RFA: Radiofrequency ablation; RPA: Recursive Partitioning Analysis; RT: Radiation therapy; RECIST: Response Evaluation Criteria in Solid Tumors; SBRT: Stereotactic body radiation therapy; SCLC: Small cell lung cancer; TACE: Trans-arterial chemo-embolization; UVA: Univariate analysis; OS: Overall survival.

## Competing interests

Dr. Brian Collins and Dr. Sean Collins are clinical consultants (including speaking) for Accuray. The remaining authors have no commercial or financial relationships that could be construed as a potential competing interest.

## Authors’ contributions

KU conceived of the study, completed chart reviews, performed statistical analysis, and helped to draft the manuscript. MK contributed to the design of the study, completed chart reviews, performed statistical analysis, and drafted the manuscript. BC, SC, AD, and GG contributed to data collection and study design. All authors read and approved the final manuscript.
